# Effect of iron supplements on cognitive development in children: an umbrella review

**DOI:** 10.3389/fnut.2026.1718507

**Published:** 2026-02-03

**Authors:** Luz Marina Caballero-Apaza, Heber Isac Arbildo-Vega, Vilma Mamani-Cori, Tania Carola Padilla-Cáceres, Fredy Hugo Cruzado-Oliva, Carlos Alberto Farje-Gallardo, Rubén Aguirre-Ipenza, Hernán Vásquez-Rodrigo, Sara Antonieta Luján-Valencia, Joan Manuel Meza-Málaga, Tania Belú Castillo-Cornock, Franz Tito Coronel-Zubiate

**Affiliations:** 1 Department of Nursing, School of Nursing, Universidad Nacional del Altiplano, Puno, Peru; 2 Faculty of Dentistry, Dentistry School, Universidad San Martín de Porres, Chiclayo, Peru; 3 Faculty of Human Medicine, Human Medicine School, Universidad San Martín de Porres, Chiclayo, Peru; 4 Faculty of Post Graduate, Universidad Nacional Toribio Rodríguez de Mendoza de Amazonas, Chachapoyas, Peru; 5 Department of General Dentistry, Dentistry School, Universidad Nacional del Altiplano, Puno, Peru; 6 Faculty of Stomatology, Stomatology School, Universidad Nacional de Trujillo, Trujillo, Peru; 7 Faculty of Health Sciences, Stomatology School, Universidad Nacional Toribio Rodríguez de Mendoza de Amazonas, Chachapoyas, Peru; 8 Faculty of Health Sciences, Universidad Continental, Lima, Peru; 9 Faculty of Health Sciences, Dentistry School, Universidad Norbert Wiener, Lima, Peru; 10 Postgraduate School, Universidad Católica de Santa María, Arequipa, Peru; 11 Faculty of Dentistry, Dentistry School, Universidad Católica de Santa María, Arequipa, Peru

**Keywords:** child, cognition, cognitive development, iron deficiency anemia, iron supplementation, neurodevelopment, umbrella review

## Abstract

**Aim:**

To synthesize and critically appraise evidence from systematic reviews and meta-analyses on the effect of iron supplementation on children’s cognitive development.

**Methods:**

We conducted a comprehensive search in PubMed, Cochrane Library, Scopus, Web of Science, Embase, and gray literature up to August 2025. Eligible studies were systematic reviews, with or without meta-analysis, assessing iron supplementation and cognitive outcomes in children. Methodological quality was appraised using AMSTAR-2, risk of bias with ROBIS, and overlap through the Corrected Covered Area (CCA). Reporting adhered to the PRIOR guidelines for overviews of reviews.

**Results:**

Of 2,725 records screened, 17 systematic reviews were included. Three reviews were rated high confidence with low risk of bias. Iron supplementation showed modest benefits in domains such as intelligence, memory, and attention among anemic children, whereas effects in non-deficient populations were negligible or uncertain. No consistent improvements were observed for broader mental development or school achievement.

**Conclusion:**

Evidence from high-confidence reviews indicates that iron supplementation confers small but meaningful cognitive benefits in anemic children, while universal supplementation in non-deficient children is not supported. These findings highlight the need for targeted supplementation strategies and more homogeneous trials to clarify long-term cognitive outcomes.

**Systematic review registration:**

doi: 10.17605/OSF.IO/5EKHX.

## 1 Introduction

Iron deficiency currently constitutes one of the leading public health problems worldwide (1). Iron deficiency anemia (IDA) is the most common cause of anemia and the most prevalent nutritional deficiency (2, 3). Children under 3 years of age are at particularly high risk due to rapid growth and, in specific subgroups such as those born with low birth weight or prematurity, reduced iron stores at birth; insufficient dietary intake further increases vulnerability (4, 5). In Europe and the United States, it is estimated that 20% of children present with iron deficiency (ID) and 5% develop IDA before the age of 3 (5, 6), while in Latin America nearly 60% of 6-month-old infants suffer from anemia (7). Beyond its impact on physical growth, iron deficiency also affects neurodevelopment and may lead to long-lasting cognitive impairment. Evidence indicates that these effects are particularly pronounced during early childhood (8–11) and may persist even after supplementation (12).

During pregnancy, iron requirements increase significantly to sustain maternal blood volume expansion and fetal transfer, exposing women to ID with or without anemia (13, 14). It is estimated that 36% of pregnant women aged 15–49 years have anemia, of which one-quarter to one-half is attributable to ID (13). Importantly, iron deficiency is not limited to low- and middle-income countries; notable prevalence levels are also reported in high-income. In Europe, the situation is particularly concerning among pregnant women, where between 28 and 85% develop iron-deficiency anemia during the third trimester (15). Oral supplementation is considered first-line therapy (16–19), yet its routine use in high-income countries is not recommended due to limited evidence of clinical benefit (16, 19). Uncertainty remains regarding optimal dosage, with recommendations ranging from 40 to 200 mg of elemental iron (20–22).

In infants, exclusive breastfeeding provides insufficient iron intake (0.35 mg/L), which may predispose to deficiency (6, 23–25). For this reason, various guidelines recommend early supplementation in breastfed infants, such as the American Academy of Pediatrics’ guideline that advises 1 mg/kg/day starting at 4 months of age (6). However, the effectiveness of this practice in healthy term infants remains debated (3, 5, 26, 27). Some studies suggest improvements in hematological and developmental parameters, as a higher risk of IDA has been reported in breastfed infants compared with formula-fed infants (28).

Biologically, iron plays a crucial role in myelination, energy metabolism, neurotransmitter synthesis, and the maturation of key brain structures such as the hippocampus and prefrontal cortex (29–32). Deficiency during sensitive periods may impair attention, memory, and processing speed. Although the literature of the past decade has strengthened the evidence base, findings remain heterogeneous. It is widely acknowledged that supplementation corrects anemia, but cognitive benefits are modest and more evident in children with initial deficiency (31, 33, 34). Moreover, neurophysiological and neuroimaging studies have reinforced the plausibility of irons role in attention and neuronal habituation (29, 35, 36).

At the cellular and molecular level, iron deficiency disrupts fundamental neurodevelopmental processes that extend beyond hematological alterations. Iron is required for mitochondrial oxidative phosphorylation, oligodendrocyte maturation, and the synthesis of monoaminergic neurotransmitters, including dopamine and serotonin, which are critical for synaptic plasticity and cortical network development (31, 32, 37, 38). Experimental and human studies suggest that iron deficiency during early life alters dendritic arborization, synaptic density, and myelin integrity, particularly in brain regions involved in executive function and memory, such as the prefrontal cortex and hippocampus (31, 32, 37). These neurobiological alterations provide a mechanistic basis for the observed vulnerability of cognitive domains to iron deficiency and help explain why supplementation appears to be most effective when administered during periods of active brain development and in children with established deficiency.

Two meta-analyses conducted in low- and middle-income countries reported small to moderate improvements in intelligence following oral supplementation, with less consistent results for memory, attention, or academic performance (33, 34). Reviews addressing maternal iron status have reported associations with cognitive and behavioral domains, although heterogeneity and variable quality limit conclusions (30, 39, 40). Findings from clinical trials are also mixed: while in healthy infants from high-income countries no benefits were observed in psychomotor development (41, 42), in Ethiopian schoolchildren intermittent iron and vitamin A supplementation improved cognitive performance (43). Additional trials have reported improvements in subscales or neurophysiological responses, though without consistent global effects (44). At the programmatic level, multiple micronutrient powders have proven effective in reducing anemia, but their cognitive benefits remain variable and dependent on baseline status and contextual factors (45–47).

On the other hand, potential risks of excessive or untargeted supplementation have been described in non-deficient populations, with contradictory findings on long-term cognitive outcomes in children fed highly fortified formulas (48). These findings highlight the importance of tailoring interventions according to risk, adjusting dosage, and considering the optimal timing of supplementation during the first 1,000 days of life (29, 31). Likewise, the coexistence of infections, inflammation, parasitic disease, and multiple nutritional deficiencies may modulate response to iron, encouraging combined micronutrient strategies and integrated public health approaches (43, 45, 46).

Despite the substantial body of evidence accumulated on ID, its implications for neurodevelopment and the effects of supplementation remain subject to debate due to heterogeneous findings across populations, socioeconomic contexts, and developmental stages (30, 33, 34, 40). Uncertainties persist regarding the optimal dosage, the most appropriate timing of supplementation, and the balance between benefits and risks, particularly in non-deficient children and pregnant women (29, 31, 48). In this context, it is necessary to systematically integrate and critically evaluate existing systematic reviews and meta-analyses, in order to assess the consistency of findings, the overall certainty of evidence, and the real impact of iron supplementation on specific domains of neurodevelopment. Accordingly, the present study aims to conduct an umbrella review that synthesizes and appraises the available evidence, identifying patterns, limitations, and gaps, and provides guidance for future research and clinical practice guidelines.

As a result, this umbrella review seeks to consolidate and interpret the current body of evidence to address the following key question: What is the effect of iron supplementation on children’s cognitive development? Additionally, this review seeks to assess the overall reliability and confidence level of the systematic reviews available on this topic.

## 2 Materials and methods

### 2.1 Protocol and registration

This umbrella review was conducted following the Preferred Reporting Items for Overviews of Reviews (PRIOR) guidelines (49). The protocol was prospectively registered in the Open Science Framework (OSF; DOI: 10.17605/OSF. IO/5EKHX), which is publicly accessible. Reporting adhered to the PRIOR checklist for overviews of reviews (50). Given the nature of the study, ethical approval was not required.

### 2.2 Eligibility criteria and results of interest

Studies eligible for inclusion were systematic reviews (with or without meta-analysis) assessing the effect of iron supplementation on children’s cognitive development.

For the purposes of this review, children were defined according to the preregistered protocol as individuals aged 2–12 years, consistent with MeSH terminology and international pediatric classifications. Reviews including broader age ranges were eligible only when the synthesized evidence pertained predominantly to this age group.

The intervention of interest was iron supplementation, administered either as a single-nutrient supplement (e.g., ferrous sulfate, ferrous fumarate) or as part of a combined formulation with additional micronutrients (e.g., iron + folic acid or multiple micronutrient powders). When systematic reviews reported mixed interventions, data specific to iron-only supplementation were extracted whenever available. When separation of effects was not possible, these reviews were included but clearly categorized as iron plus co-supplementation.

Comparator groups included placebo, no supplementation, or usual diet, as reported in the synthesized primary studies. Reviews in which the comparator group involved iron administration were excluded.

Eligible studies were required to report outcomes related to cognitive or neurodevelopmental performance, including but not limited to: global cognitive development, intelligence, attention, memory, executive functions, mental development indices, neurobehavioral measures, or school achievement. Reviews focusing exclusively on hematologic or growth outcomes were excluded unless cognitive outcomes were also analyzed.

No restrictions were applied regarding publication date or language. Exclusion criteria were narrative or rapid reviews, interventional studies, observational research, preclinical and basic science studies, protocols, abstracts, case reports, commentaries, letters, opinions, and poster presentations.

### 2.3 Sources of information, search strategy and additional search for primary studies

An electronic literature search was conducted covered records up to August 19, 2025, using six major databases: PubMed, Cochrane Library, Scopus, Web of Science, Embase and SciELO. These databases were predefined in the preregistered protocol (OSF DOI: 10.17605/OSF. IO/5EKHX). To identify additional records, gray literature sources were also consulted, including Google Scholar, ProQuest Dissertations and Theses, and OpenGrey. Reference lists of included studies were manually screened to identify any relevant additional publications. All retrieved articles were imported into Zotero^®^ (Center for History and New Media, Virginia, United States), and duplicates were removed. Detailed search strategies for each database are presented in Supplementary material S1. Studies published after the search cut-off date (19 August 2025) were not eligible for inclusion, in accordance with the preregistered protocol.

### 2.4 Data management and selection process

The screening and selection process was performed in two stages using Rayyan^®^ online software (Qatar Computing Research Institute, Qatar). In the first phase, two independent reviewers (L. C. and T. P.) assessed titles and abstracts. In the second phase, the full texts of potentially relevant studies were evaluated independently by the same reviewers. Disagreements at any stage were resolved through discussion with a third reviewer (H. A.).

### 2.5 Data collection process

Data extraction was carried out independently and in duplicate by two reviewers (V. M. and F. C. O.) using a standardized data collection form. Extracted information was cross-checked for consistency, and disagreements were resolved by a third author (H. A), who acted as adjudicator. The following variables were recorded: author names, year of publication, type of systematic review, characteristics of included primary studies, number of studies included in qualitative and quantitative analyses, reported outcomes, main conclusions, and whether the reviews reported adherence to PRISMA guidelines, PROSPERO registration or another public platform, use of the GRADE system, and performance of a meta-analysis.

### 2.6 Assessment of methodological quality, quality of evidence and meta-bias

The methodological quality of included reviews was assessed independently and in duplicate by two reviewers (F. C. Z. and R. A.), with inter-rater agreement (Kappa = 0.85). We applied the AMSTAR-2 tool (51) to evaluate methodological quality and the ROBIS tool (52) to assess risk of bias. The overall confidence level was categorized as high, moderate, low, or critically low; risk of bias as low, unclear, or high. To evaluate overlap of primary studies across reviews, the Corrected Covered Area (CCA) index was calculated.

### 2.7 Summary of measures

For systematic reviews without meta-analysis, outcomes were extracted and synthesized narratively. When meta-analyses were available, results were recorded as mean differences (MD) or standardized mean differences (SMD), with 95% confidence intervals.

### 2.8 Summary of results

The primary findings were synthesized by cognitive domains, including cognitive development, mental development, intelligence, attention and concentration, memory, and school achievement.

## 3 Results

### 3.1 Identification and selection of studies

**Figure 1:**
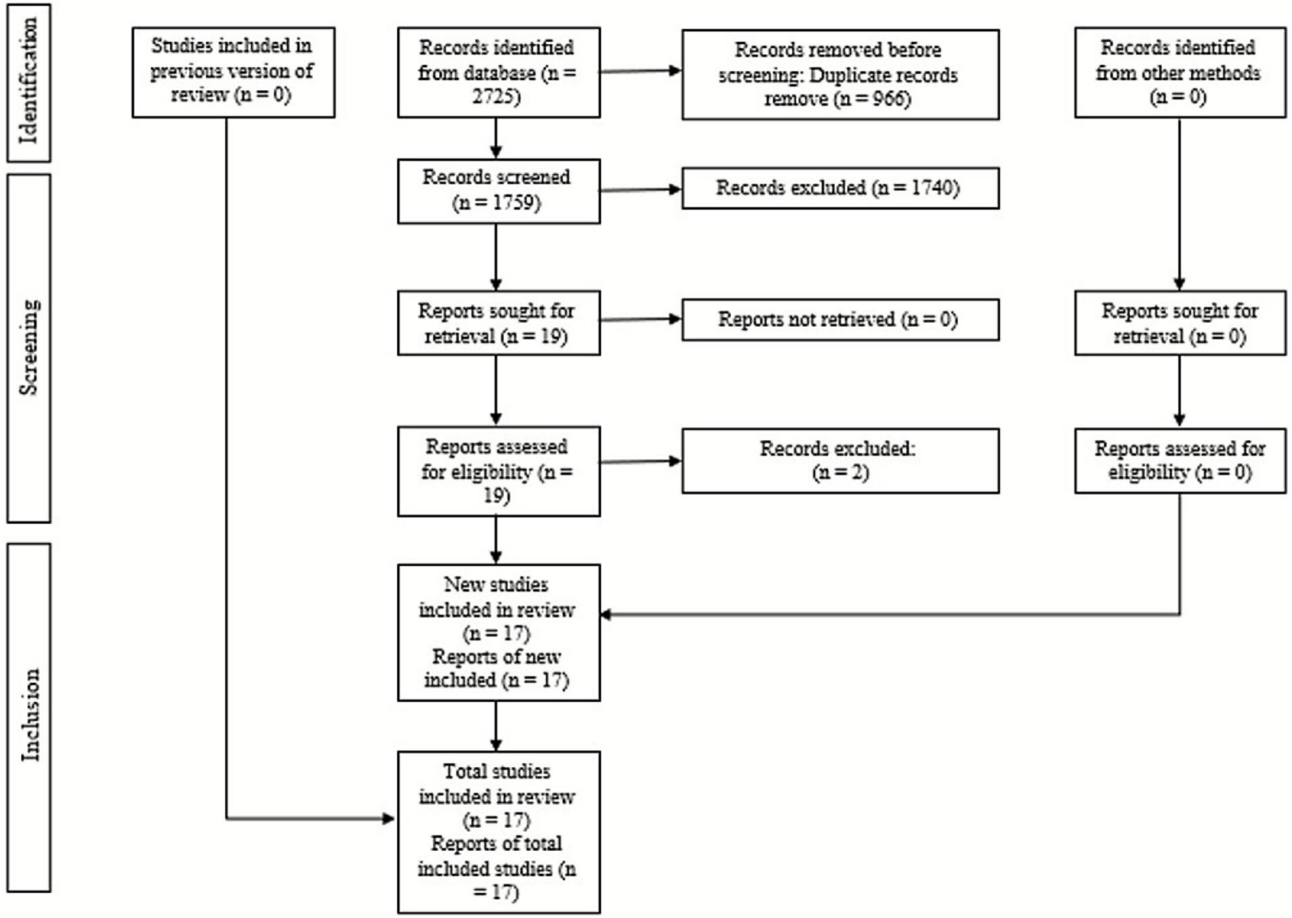
PRISMA flow diagram showing the selection process of studies included in the systematic review, from initial identification to final inclusion.

A total of 2,725 records were identified through the electronic search. After removing duplicates, 1,759 unique references remained. Following title/abstract screening, 19 studies were evaluated in full text, of which 2 were excluded. Seventeen systematic reviews (SRs) were therefore included. Reasons for exclusion are detailed in Supplementary material S2. The study selection process is shown in the PRISMA flow diagram (Figure 1).

### 3.2 Characteristics of included studies

The included systematic reviews (SRs) were published between 2005 and 2025, and originated from a wide range of countries, including China (53–56), Australia (40, 57–59), Ethiopia (33), Belgium (33), Kenya (60), United Kingdom (31, 60), Gambia (31), Canada (61, 62), Switzerland (63), United State (63, 64), Germany (65), Serbia (65), Spain (65), Poland (66), and India (64).

All SRs evaluated the effects of iron supplementation on cognitive outcomes in children and met our prespecified eligibility criteria. Across reviews, the populations consistently fell within the pediatric age range established in our protocol (2–12 years), although individual SRs synthesized heterogeneous primary studies with varying age distributions. Most SRs did not report the proportion of children with anemia, as this information was inconsistently available in the underlying primary studies.

Further details regarding the characteristics of the SRs are provided in Supplementary material S3.

### 3.3 Methodological quality and risk of bias

Three systematic reviews (31, 40, 53) were considered to have high confidence, 8 SRs (33, 56, 58–60, 62, 63, 65) they had low confidence and 6 SRs (54, 55, 57, 61, 64, 66) had critically low confidence (Supplementary material S4). Furthermore, 2 SRs (54, 55) were found to have a high risk of bias, 7 SRs (58, 61–66) were found to have an unclear risk of bias, and 8 SRs (31, 33, 40, 53, 56, 57, 59, 60) were found to have a low risk of bias (Supplementary material S3).

To enhance robustness, a sensitivity analysis excluding low/critically low confidence SRs and those at high/unclear risk of bias was performed. However, while all included SRs are described in the Results section, the interpretative synthesis that supports the main conclusions is based primarily on high-confidence, low-risk SRs.

### 3.4 Overlap of primary studies

A total of 119 primary studies were identified across SRs. The Corrected Covered Area (CCA) was 4.69%, indicating slight overlap. Specifically, 12 studies appeared twice, 5 three times, 5 four times, 2 five times, and 1 was included in seven SRs. Further details are provided in Supplementary material S5.

### 3.5 Evidence synthesis

The main results are summarized in Supplementary material S3. Below, findings are presented by cognitive domain.

### 3.6 Cognitive development

Four SRs (54, 57, 58, 65) included reported no positive or negative effect of iron supplementation on children’s cognitive development, while 4 SRs (31, 40, 55, 60) reported a positive effect. Five SRs (54, 55, 57, 58, 60) conducted meta-analyses, reporting MD ranging values from 0.25 [95% CI: 0.06, 0.45] (57) to 1.73 [95% CI: −1.05, 4.52] (60) and the SMD ranged from 0.09 [95 CI: 0.03, 0.15] (54) to 0.5 [95% CI: 0.11, 0.9] (58).

One SR (60) included reported no positive or negative effect of iron supplementation on anemic children’s cognitive development and another SR (58) reported the same in non-anemic children, while one SR (58) included reported a positive effect. In subgroup analyses, the pooled MD for anemic children was −7.20 [95% CI: −21.58, 7.18] (60), with an SMD of 0.29 [95% CI: 0.07, 0.51] (58), whereas in non-anemic children the SMD was SMD 0.01 [95% CI: −0.1, 0.11] (58).

Moumin et al. (40) and McCann et al. (31) presented the results descriptively and reported higher doses of prenatal iron, compared with lower doses, neither benefited nor harmed cognitive development in infants younger than 24 months; however, an improvement was observed in children aged 6–59 months. Hermoso et al. (65) reported an improvement in the relationship between iron supplementation and cognitive development in children.

### 3.7 Attention and concentration

One SR (33) reported a positive effect of iron supplementation on attention and concentration in children, but no positive or negative effect of iron supplementation only. In this review, “iron supplementation only” refers to iron administered as a single-nutrient supplement, in contrast to interventions combining iron with other micronutrients (e.g., vitamin A or multiple micronutrient powders). In the meta-analysis, the SMD for iron supplementation was 0.45 [95% CI: 0.09, 0.8] and for iron given alone (single-nutrient supplementation) it was 0.33 [95% CI: −0.39, 1.05].

### 3.8 Intelligence

One SR (33) included reported a positive effect of iron supplementation on intelligence in children, while one SR (58) included reported no positive or negative effect. In pooled analyses, the SMD was 0.46 [95% CI: 0.19, 0.73] (33) and the MD was 4.58 [95% CI: −2.5 to 11.66] (58).

One SR (33) reported no positive or negative effect of iron supplementation only on intelligence in children. The corresponding meta-analysis yielded an SMD of 0.2 [95% CI: −0.04, 0.44].

Two SRs (33, 58) included reported a positive effect of iron supplementation on anemic children’s intelligence, but no positive or negative effect on non-anemic children. For anemic children, pooled estimates showed an SMD of 0.79 [95% CI: 0.41, 1.16] (33) and a MD of 4.55 [95% CI: 0.16, 8.94] (58), whereas for non-anemic children was SMD 0.01 [95% CI: −0.1, 0.12] (33) and MD was 0.08 [95% CI: −1.86, 2.01] (58).

### 3.9 Memory

One SR (33) reported a positive effect of iron supplementation on memory in children, the same in anemic children and effect of iron supplementation only, but no positive or negative effect in non-anemic children. In this meta-analysis, the SMD for iron supplementation was 0.45 [95% CI: 0.04, 0.69], for iron given alone it was 0.33 [95% CI: 0.08, 0.57]. Among anemic children, the SMD was 0.47 [95% CI: 0.13, 0.81], whereas in non-anemic children it was 0.02 [95% CI: −1.01, 0.97].

### 3.10 Mental development

Five SRs (53, 59, 61, 63, 66) included reported no positive or negative effect of iron supplementation on children’s mental development, while 2 SRs (62, 64) reported a positive effect. All these SRs included meta-analyses, with MD estimates ranging from −0.93 [95% CI: −3.87, 2.02] (53) to 4.14 [95% CI: 0.1, 8.18] (62) and an SMD of 0.30 [95% CI: 0.15, 0.46] (64).

One SR (64) included reported a positive effect of iron supplementation on anemic children’s mental development, while 2 SRs (56, 59) included reported no positive or negative effect. For anemic children, pooled MD values ranged from 1.04 [95% CI: −1.3, 3.39] (56) to 4.46 [95%CI: −9.32, 18.24] (59), with an SMD of 0.5 [95% CI: 0.25, 0.75] (64). For non-anemic children, the MD was 1.49 [95% CI: −1.08, 4.07] (59) and the SMD was −0.11 [95% CI: −0.36, 0.14] (64).

### 3.11 School achievement

One SR (33) reported no positive or negative effect of iron supplementation on children’s school achievement, the same in anemic or non-anemic children. In the meta-analysis, the SMD for children’s school achievement overall was 0.00 [95% CI: −0.21, 0.21]; among anemic children it was 0.12 [95% CI: −0.63, 0.39], and among non-anemic children 0.43 [95% CI: −0.74, 1.59].

## 4 Discussion

Scientific interest in the relationship between iron supplementation and child cognitive development has grown significantly over the past decades, given that ID is one of the most prevalent nutritional deficiencies worldwide and its impact on neurodevelopment is potentially irreversible (31, 37, 38). Randomized controlled trials (RCTs) have shown heterogeneous results: while some studies report improvements in cognitive parameters after supplementation, particularly in children with anemia (33, 37, 55), others have not demonstrated clear benefits in healthy populations or in high-income countries (41). Likewise, previous SRs have indicated a pattern of inconsistent findings, with modest benefits depended on baseline iron status (40, 65). In addition, the systematic reviews included in this umbrella review encompassed children with heterogeneous baseline iron status and anemia risk, ranging from iron-deficient to apparently healthy populations. This broad inclusion reflects real-world practice and is consistent with World Health Organization recommendations, which endorses both therapeutic and preventive iron supplementation in settings with a high burden of anemia and among children at increased risk (67).

Our study, by integrating evidence from 17 SRs, provides a comprehensive and updated overview. A key strength lies in the use of a sensitivity analysis that included only high-confidence reviews with low risk of bias, thus providing greater robustness to the conclusions. However, certain limitations must be acknowledged: methodological heterogeneity among primary studies, variability in supplementation doses and duration, as well as the diversity of cognitive tests employed, simplified phrasing of results. Moreover, although the degree of overlap of primary studies was low (CCA: 4.69%), partial repetition of some studies may have influenced the weight of certain findings.

### 4.1 Evidence summary

Overall, the results show inconsistent effects of iron supplementation on cognitive development, reflecting the complex and context-dependent role of iron in brain maturation. While four SRs reported no effect on global cognitive outcomes (54, 57, 58, 65), another four identified benefits, particularly in specific populations or cognitive domains (31, 40, 55, 60). In children with anemia, some studies reported significant improvements in cognitive development, consistent with previous findings that emphasize the importance of baseline iron status as a modulator of response (37, 55). A possible explanation is that iron, being essential for myelination, synaptogenesis and neurotransmitter synthesis, improves cognitive functions primarily when deficiency is present; in healthy children, where iron stores are already adequate for neurodevelopmental processes, additional supplementation does not produce detectable changes (4, 31, 32, 37, 38).

From a biological perspective, the heterogeneous cognitive effects observed across studies can be partly explained by the molecular and cellular consequences of iron deficiency on the developing central nervous system. Iron is essential for mitochondrial energy metabolism, oligodendrocyte function, myelination, synaptogenesis, and neurotransmitter synthesis; deficiency during critical developmental windows disrupts these processes, particularly in brain regions such as the hippocampus and prefrontal cortex that underlie memory, attention, and executive functions (31, 32, 37, 38). When iron deficiency is present, supplementation may restore altered cellular pathways and neural signaling, leading to measurable cognitive improvements. In contrast, in iron-replete children, neurodevelopmental processes may already be operating near their physiological capacity, and additional iron intake does not confer further neurocognitive benefit, which may explain the absence of consistent effects in healthy populations and high-income settings (31–34, 37).

With respect to attention and concentration, one review reported a positive effect, although this benefit was not consistently observed when iron supplementation alone was analyzed. This may be because attention is a function highly sensitive to iron availability in the prefrontal cortex, but also dependent on other micronutrients and environmental factors (31, 32, 37, 38). Therefore, benefits may only be evident in contexts where deficiency is severe or coexists with other deficiencies.

For intelligence, the results suggest a favorable impact in anemic children, but not in non-anemic children. This aligns with the hypothesis that iron acts as a limiting nutrient only in deficiency states, and that in the absence of deficiency, cognitive functions are not enhanced by additional intake (33, 65). The lack of effect in non-anemic children may also be explained by a “biological ceiling,” i.e., saturation of neurocognitive processes when iron availability is already adequate.

Regarding memory, moderate improvements were reported, particularly in anemic children, while no significant differences were observed in non-anemic groups. The likely explanation is that iron plays a key role in hippocampal function and myelination, which are central to memory consolidation, so deficiency directly affects this domain (31, 32, 37, 38). However, when levels are normal, other factors such as environmental stimulation, overall nutritional status, and educational quality may weigh more heavily on memory performance.

In mental development, most studies did not find benefits, although 2 reviews reported positive effects, mainly in populations with prior deficiency (64). This may be because mental development is a broader and multifactorial construct that includes cognitive, emotional and social skills, and does not depend exclusively on iron (8, 10, 11, 14, 31, 32, 37). Thus, the effect of supplementation may be diluted when psychosocial and educational determinants are not addressed concurrently.

Finally, no significant improvements were observed in school achievement. This finding can be understood considering that academic performance is strongly influenced by external variables such as teaching quality, family environment, and socioeconomic status (8, 10, 11, 14, 31, 32, 37, 39). In this sense, although iron may improve certain neurocognitive parameters, its isolated impact would not be sufficient to translate into tangible academic gains without complementary educational interventions.

Taken together, the heterogeneity of results may be explained by the interaction of biological factors (baseline iron status, age, and critical stages of neurodevelopmental), methodological factors (variability in dosage, duration of supplementation, and cognitive tests applied), and contextual factors (socioeconomic conditions, early stimulation, coexistence of multiple deficiencies). This complexity suggests that the effect of iron on cognition is not uniform, but depends on critical developmental windows, nutritional and social context, and the methodological rigor of the studies. In addition, this umbrella review necessarily combines evidence from populations with very different baseline iron status and risk profiles, which, although it provides a broad global picture, may oversimplify conclusions if not interpreted with caution. The substantial variation in the methodological quality of the included reviews also remains an important limitation, even though our main conclusions are based on high-confidence and low–risk-of-bias reviews. In addition, the marked differences in anemia prevalence and in the observed cognitive effects of iron supplementation between countries in the Global North and the Global South are unlikely to be explained by economic factors alone. A growing body of evidence indicates that these disparities are shaped by a complex interplay of biological, environmental, and health-system determinants. These include a higher burden of infectious and parasitic diseases, chronic inflammation affecting iron absorption and utilization, differences in dietary patterns and iron bioavailability, maternal nutritional status, and limited access to early screening and preventive care. Furthermore, variations in national fortification policies, supplementation strategies, and timing of interventions during critical developmental windows may contribute to heterogeneous outcomes across regions. This broader contextual framework helps explain why iron supplementation tends to show more consistent cognitive benefits in high-burden settings, while effects are less evident in low-prevalence contexts.

### 4.2 Implications for clinical practice

The results of this umbrella review confirm that the benefits of iron supplementation on cognitive development are context-dependent, with clearer effects in children with anemia or at high risk of deficiency. Therefore, universal supplementation in healthy children is not justified, since benefits in domains such as intelligence, memory, or school performance are scarce and inconsistent, while the risk of overdosing or adverse effects remains present (33, 48).

In pediatric clinical practice, these findings reinforce the importance of implementing early screening strategies for iron status, especially in infants with low birth weight, deficient diets, or a history of anemia. In these groups, targeted supplementation can prevent cognitive deficits during sensitive periods of neurodevelopment. Conversely, in children without risk factors or with adequate iron stores, routine supplementation should be avoided, favoring an approach based on clinical monitoring and family nutrition education.

At the public health level, the results suggest that supplementation programs should prioritize vulnerable populations, such as infants in regions with high anemia prevalence, schoolchildren in rural areas with limited food access, and pregnant women, in whom ID can affect both maternal health and child neurodevelopment (48, 57). This is particularly relevant in low- and middle-income countries, where the burden of IDA is higher and the potential benefits of supplementation may be more tangible.

In light of the heterogeneity observed across the included systematic reviews—many of which were conducted in low- and middle-income settings—it is likely that the response to iron supplementation does not occur in isolation, but within a context characterized by frequent infections, parasitic diseases and concurrent deficiencies of other micronutrients (7, 43–47, 60, 63). In such contexts, several primary trials and reviews have evaluated iron as part of multiple micronutrient powders or in combination with other interventions, suggesting that multidimensional strategies may yield broader benefits on anemia and selected developmental outcomes than iron alone (43–47, 63). These considerations, while going beyond the direct comparisons synthesized in our umbrella review, provide important contextual background for interpreting our findings and designing integrated child health programs.

### 4.3 Implications for research

The findings of this umbrella review highlight several knowledge gaps that must be addressed in future research. First, the heterogeneity observed across studies underscores the need for more homogeneous RCTs, with standardized protocols regarding dosage, duration of supplementation, and age at initiation onset. The large variability in these aspects limits comparability and hampers the establishment of robust conclusions on the magnitude of iron’s effect on neurodevelopment.

Second, future studies should employ validated and uniform instruments to assess cognitive domains (e.g., memory, attention, intelligence), since the use of heterogeneous tests has contributed to inconsistent results. Likewise, studies should report differentiated effects in children with anemia versus those without, as the findings of this review clearly show distinct responses between these groups.

Another crucial aspect is the need for longitudinal trials evaluating the long-term impact of iron supplementation, from childhood to adolescence. This would help determine whether early improvements in neurocognitive parameters translate into sustained benefits, such as better academic performance, executive skills, or productivity in adulthood.

Furthermore, it is essential to investigate interactions between iron and other micronutrients, as well biological factors such as infections or chronic inflammation, which may modulate cognitive outcomes of supplementation. In this regard, future studies should consider multifactorial designs including combined interventions, more representative of real-world public health contexts.

Finally, future SRs should incorporate more rigorous risk of bias and quality evidence assessments, as well as subgroup analyses by region, socioeconomic status, and nutritional background. The use of modern tools such as biomarkers of iron status, neuroimaging, and neurophysiological techniques (e.g., evoked potentials) could help elucidate underlying mechanisms and provide mechanistic evidence to complement clinical findings.

## 5 Conclusion

The evidence synthesized in this umbrella review indicates that the effects of iron supplementation on child cognitive development are heterogeneous and strongly influenced on baseline iron status, age, and socioeconomic context. Benefits are more consistent in children with anemia or iron deficiency, with modest improvements observed in intelligence, memory, and attention, whereas in non-deficient children the effects are negligible or uncertain. The lack of impact on broader domains such as overall mental development and school achievement reinforces the notion that while iron is essential for key neurobiological processes, including myelination, synaptogenesis, energy metabolism, and neurotransmitter synthesis in the developing brain, it does not act in isolation on cognitive and academic performance but rather in interacts with nutritional, psychosocial, and environmental factors.

By integrating clinical outcomes with current mechanistic evidence from neurobiological and neurophysiological studies, these findings highlight the importance of prioritizing targeted supplementation strategies for vulnerable and high-risk populations rather than universal interventions in healthy children. They also emphasize the need for more rigorous research, with homogeneous study designs, long-term evaluations, and the incorporation of biomarkers and neurophysiological tools to clarify underlying mechanisms. Taken together, the available evidence suggests that iron is an important but not exclusive determinant of neurodevelopment, and that its clinical impact should be interpreted within an integrated framework of child health and public health.

## Data Availability

The original contributions presented in the study are included in the article/Supplementary material, further inquiries can be directed to the corresponding author.
