# Supplementary material for: Effect of iron supplements on cognitive development in children: an umbrella review
Source: Front Nutr. 2026 Feb 3;13:1718507. doi: 10.3389/fnut.2026.1718507 (PMC12909201; doi:10.3389/fnut.2026.1718507)
Supplement: Supplementary file 1 [file Table_1.docx]

Supplementary Material 1. Database search strategy

| **Database** | **Search strategy** | **Number of studies** |
| --- | --- | --- |
| Pubmed | ("cognition"[MeSH Terms] OR "cognition"[Title/Abstract] OR "cognitive"[Title/Abstract] OR "child development"[MeSH Terms] OR "child develop*"[Title/Abstract] OR "language develop*"[Title/Abstract] OR "language learn*"[Title/Abstract] OR "language train*"[Title/Abstract] OR "language acquisition"[Title/Abstract] OR "intelligence tests"[MeSH Terms] OR "intelligence test*"[Title/Abstract] OR "intelligence measurement*"[Title/Abstract] OR "intelligence"[MeSH Terms] OR "intelligen*"[Title/Abstract] OR "neuropsychological tests"[MeSH Terms] OR "neuropsychological test*"[Title/Abstract] OR "neuropsychologic assessment*"[Title/Abstract] OR "neuropsychologic examination*"[Title/Abstract] OR "wechsler scale*"[Title/Abstract] OR "wisc v"[Title/Abstract] OR "wisc iv"[Title/Abstract] OR "wais r"[Title/Abstract] OR "wppsi"[Title/Abstract] OR "binet test*"[Title/Abstract] OR "psychology, developmental"[MeSH Terms] OR "developmental psychology"[Title/Abstract] OR "academic success"[MeSH Terms] OR "academic success*"[Title/Abstract] OR "achievement"[MeSH Terms] OR "academic achievement*"[Title/Abstract] OR "education* success*"[Title/Abstract] OR "education* achievement*"[Title/Abstract] OR "academic performance"[MeSH Terms] OR "academic performance*"[Title/Abstract] OR "academic test* score*"[Title/Abstract] OR "educational performance*"[Title/Abstract] OR "educational test* score*"[Title/Abstract] OR "learning curve"[MeSH Terms] OR "learning curve"[Title/Abstract] OR "psychomotor performance"[MeSH Terms] OR "psychomotor performance*"[Title/Abstract] OR "visual motor performance*"[Title/Abstract] OR "visuomotor coordination"[Title/Abstract] OR "perceptual motor performance"[Title/Abstract] OR "aptitude tests"[MeSH Terms] OR "aptitude test*"[Title/Abstract] OR "multitasking behavior"[MeSH Terms] OR "multitask* behavior*"[Title/Abstract] OR "underachievement"[MeSH Terms] OR "underachiev*"[Title/Abstract] OR "executive function"[MeSH Terms] OR "executive function*"[Title/Abstract] OR "executive control*"[Title/Abstract] OR "learning"[MeSH Terms] OR "learning"[Title/Abstract] OR "learn"[Title/Abstract] OR "learnings"[Title/Abstract] OR "learns"[Title/Abstract] OR "problem solving"[MeSH Terms] OR "problem solving"[Title/Abstract] OR "thinking"[MeSH Terms] OR "thinking"[Title/Abstract]) AND ("iron"[MeSH Terms] OR "iron"[Title/Abstract] OR "ferric compounds"[MeSH Terms] OR "ferric*"[Title/Abstract] OR "ferrous compounds"[MeSH Terms] OR "ferrous*"[Title/Abstract] OR "fe"[Title/Abstract] OR "anemia"[MeSH Terms] OR "anemia*"[Title/Abstract] OR "anaemia*"[Title/Abstract] OR "iron binding proteins"[MeSH Terms] OR "dietary supplements"[MeSH Terms] OR "dietary supplement*"[Title/Abstract] OR "diet"[MeSH Terms] OR "diet supplement*"[Title/Abstract] OR "minerals"[MeSH Terms] OR "minerals supplement*"[Title/Abstract] OR "mineral supplement*"[Title/Abstract] OR "multinutrient supplement*"[Title/Abstract] OR "micronutrients"[MeSH Terms] OR "micronutrient* supplement*"[Title/Abstract] OR "trace element* supplement*"[Title/Abstract]) AND ("child"[MeSH Terms] OR "child"[Title/Abstract] OR "children"[Title/Abstract] OR "childrens"[Title/Abstract] OR "childs"[Title/Abstract] OR "boy"[Title/Abstract] OR "boys"[Title/Abstract] OR "boyhood"[Title/Abstract] OR "girl"[Title/Abstract] OR "girls"[Title/Abstract] OR "girlhood"[Title/Abstract]) AND ("systematic review"[Title/Abstract] OR "meta-analysis"[Title/Abstract]) | 390 |
| Cochrane database | #1 MeSH descriptor: [Cognition] explode all trees  #2 MeSH descriptor: [Cognitive Neuroscience] explode all trees  #3 MeSH descriptor: [Child Development] explode all trees  #4 MeSH descriptor: [Language Development] explode all trees  #5 MeSH descriptor: [Intelligence Tests] explode all trees  #6 MeSH descriptor: [Intelligence] explode all trees  #7 MeSH descriptor: [Neuropsychological Tests] explode all trees  #8 MeSH descriptor: [Wechsler Scales] explode all trees  #9 MeSH descriptor: [Stanford-Binet Test] explode all trees  #10 MeSH descriptor: [Psychology, Developmental] explode all trees  #11 MeSH descriptor: [Academic Success] explode all trees  #12 MeSH descriptor: [Achievement] explode all trees  #13 MeSH descriptor: [Academic Performance] explode all trees  #14 MeSH descriptor: [Learning Curve] explode all trees  #15 MeSH descriptor: [Psychomotor Performance] explode all trees  #16 MeSH descriptor: [Aptitude Tests] explode all trees  #17 MeSH descriptor: [Multitasking Behavior] explode all trees  #18 MeSH descriptor: [Underachievement] explode all trees  #19 MeSH descriptor: [Learning] explode all trees  #20 MeSH descriptor: [Verbal Learning] explode all trees  #21 MeSH descriptor: [Serial Learning] explode all trees  #22 MeSH descriptor: [Memory and Learning Tests] explode all trees  #23 MeSH descriptor: [Problem Solving] explode all trees  #24 MeSH descriptor: [Thinking] explode all trees  #25 ("cognition"):ti,ab,kw OR ("cognitive"):ti,ab,kw OR ("child development"):ti,ab,kw OR ("language development"):ti,ab,kw OR ("language learning"):ti,ab,kw OR ("language training"):ti,ab,kw OR ("language acquisition"):ti,ab,kw OR ("intelligence test"):ti,ab,kw OR ("intelligence measurement"):ti,ab,kw OR ("intelligence"):ti,ab,kw OR ("intelligence quotient"):ti,ab,kw OR ("neuropsychological test"):ti,ab,kw OR ("neuropsychologic assessment"):ti,ab,kw OR ("neuropsychologic examination"):ti,ab,kw OR ("wechsler scale"):ti,ab,kw OR ("wms iv nl"):ti,ab,kw OR ("wisc v"):ti,ab,kw OR ("wisc iv"):ti,ab,kw OR ("wais r"):ti,ab,kw OR ("wechsler preschool and primary scale of intelligence"):ti,ab,kw OR ("wppsi"):ti,ab,kw OR ("binet test"):ti,ab,kw OR ("developmental psychology"):ti,ab,kw OR ("academic success"):ti,ab,kw OR ("academic achievement"):ti,ab,kw OR ("education success"):ti,ab,kw OR ("education achievement"):ti,ab,kw OR ("academic performance"):ti,ab,kw OR ("academic test score"):ti,ab,kw OR ("educational performance"):ti,ab,kw OR ("educational test score"):ti,ab,kw OR ("educational test performance"):ti,ab,kw OR ("learning curve"):ti,ab,kw OR ("psychomotor performance"):ti,ab,kw OR ("visual motor performance"):ti,ab,kw OR ("visuomotor coordination"):ti,ab,kw OR ("perceptual motor performance"):ti,ab,kw OR ("aptitude test"):ti,ab,kw OR ("multitasking behavior"):ti,ab,kw OR ("underachievement"):ti,ab,kw OR ("learning"):ti,ab,kw OR ("learn"):ti,ab,kw OR ("learnings"):ti,ab,kw OR ("learns"):ti,ab,kw OR ("problem solving"):ti,ab,kw OR ("thinking"):ti,ab,kw  #26 #1 OR #2 OR #3 OR #4 OR #5 OR #6 OR #7 OR #8 OR #9 OR #10 OR #11 OR #12 OR #13 OR #14 OR #15 OR #16 OR #17 OR #18 OR #19 OR #20 OR #21 OR #22 OR #23 OR #24 OR #25  #27 MeSH descriptor: [Child] explode all trees  #28 ("child"):ti,ab,kw OR ("children"):ti,ab,kw OR ("childrens"):ti,ab,kw OR ("childs"):ti,ab,kw OR ("boy"):ti,ab,kw OR ("boys"):ti,ab,kw OR ("boyhood"):ti,ab,kw OR ("girl"):ti,ab,kw OR ("girls"):ti,ab,kw OR ("girlhood"):ti,ab,kw  #29 #27 OR #29  #30 MeSH descriptor: [Iron] in all MeSH products  #31 MeSH descriptor: [Iron, Dietary] explode all trees  #32 MeSH descriptor: [Ferric Compounds] explode all trees  #33 MeSH descriptor: [Ferrous Compounds] explode all trees  #34 MeSH descriptor: [Anemia] explode all trees  #35 MeSH descriptor: [Anemia, Iron-Deficiency] explode all trees  #36 MeSH descriptor: [Iron-Binding Proteins] explode all trees  #37 MeSH descriptor: [Dietary Supplements] explode all trees  #38 MeSH descriptor: [Diet] explode all trees  #39 MeSH descriptor: [Minerals] in all MeSH products  #40 MeSH descriptor: [Micronutrients] explode all trees  #41 MeSH descriptor: [Trace Elements] explode all trees  #42 ("iron"):ti,ab,kw OR ("ferric"):ti,ab,kw OR ("ferrous"):ti,ab,kw OR ("fe"):ti,ab,kw OR ("anemia"):ti,ab,kw OR ("anaemia"):ti,ab,kw OR ("iron-deficiency"):ti,ab,kw OR ("dietary supplement"):ti,ab,kw OR ("diet supplement"):ti,ab,kw OR ("minerals supplement"):ti,ab,kw OR ("mineral supplement"):ti,ab,kw OR ("multinutrient supplement"):ti,ab,kw OR ("multinutrients supplement"):ti,ab,kw OR ("micronutrient supplement"):ti,ab,kw OR ("trace element supplement"):ti,ab,kw  #43 #30 OR #31 OR #32 OR #33 OR #34 OR #35 OR #36 OR #37 OR #38 OR #39 OR #40 OR #41 OR #42  #44 #26 AND #29 AND #43 | 44 |
| Scopus | TITLE-ABS-KEY(("cognition" OR "cognitive" OR "child development" OR "language development" OR "language learning" OR "language training" OR "language acquisition" OR "intelligence tests" OR "intelligence measurement" OR "intelligence" OR "neuropsychological tests" OR "neuropsychologic assessment" OR "neuropsychologic examination" OR "wechsler scale" OR "wisc v" OR "wisc iv" OR "wais r" OR "wppsi" OR "binet test" OR "developmental psychology" OR "academic success" OR "achievement" OR "academic achievement" OR "education success" OR "education achievement" OR "academic performance" OR "academic test score" OR "educational performance" OR "educational test score" OR "learning curve" OR "psychomotor performance" OR "visual motor performance" OR "visuomotor coordination" OR "perceptual motor performance" OR "aptitude test" OR "multitasking behavior" OR "underachievement" OR "executive function" OR "executive control" OR "learning" OR "learn" OR "learnings" OR "learns" OR "problem solving" OR "thinking")) AND TITLE-ABS-KEY(("iron" OR "ferric compounds" OR "ferric" OR "ferrous compounds" OR "ferrous" OR "fe" OR "anemia" OR "anaemia" OR "iron binding proteins" OR "dietary supplement" OR "diet" OR "diet supplement" OR "minerals" OR "mineral supplement" OR "multinutrient supplement" OR "micronutrient" OR "micronutrient supplement" OR "trace element supplement")) AND TITLE-ABS-KEY(("child" OR "children" OR "childrens" OR "childs" OR "boy" OR "boys" OR "boyhood" OR "girl" OR "girls" OR "girlhood")) AND TITLE-ABS-KEY(("systematic review" OR "meta-analysis")) | 1101 |
| Web of Science | TS=(("cognition" OR "cognitive" OR "child development" OR "language development" OR "language learning" OR "language training" OR "language acquisition" OR "intelligence tests" OR "intelligence measurement" OR "intelligence" OR "neuropsychological tests" OR "neuropsychologic assessment" OR "neuropsychologic examination" OR "wechsler scale" OR "wisc v" OR "wisc iv" OR "wais r" OR "wppsi" OR "binet test" OR "developmental psychology" OR "academic success" OR "achievement" OR "academic achievement" OR "education success" OR "education achievement" OR "academic performance" OR "academic test score" OR "educational performance" OR "educational test score" OR "learning curve" OR "psychomotor performance" OR "visual motor performance" OR "visuomotor coordination" OR "perceptual motor performance" OR "aptitude test" OR "multitasking behavior" OR "underachievement" OR "executive function" OR "executive control" OR "learning" OR "learn" OR "learnings" OR "learns" OR "problem solving" OR "thinking") AND ("iron" OR "ferric compounds" OR "ferric" OR "ferrous compounds" OR "ferrous" OR "fe" OR "anemia" OR "anaemia" OR "iron binding proteins" OR "dietary supplement" OR "diet" OR "diet supplement" OR "minerals" OR "mineral supplement" OR "multinutrient supplement" OR "micronutrient" OR "micronutrient supplement" OR "trace element supplement") AND ("child" OR "children" OR "boy" OR "boys" OR "girl" OR "girls") AND ("systematic review" OR "meta-analysis")) | 851 |
| Embase | ('cognition':ti,ab,kw OR 'cognitive':ti,ab,kw OR 'child development':ti,ab,kw OR 'language development':ti,ab,kw OR 'language learning':ti,ab,kw OR 'language training':ti,ab,kw OR 'language acquisition':ti,ab,kw OR 'intelligence test':ti,ab,kw OR 'intelligence measurement':ti,ab,kw OR 'intelligence':ti,ab,kw OR 'intelligence quotient':ti,ab,kw OR 'neuropsychological test':ti,ab,kw OR 'neuropsychologic assessment':ti,ab,kw OR 'neuropsychologic examination':ti,ab,kw OR 'wechsler scale':ti,ab,kw OR 'wms iv nl':ti,ab,kw OR 'wisc v':ti,ab,kw OR 'wisc iv':ti,ab,kw OR 'wais r':ti,ab,kw OR 'wechsler preschool and primary scale of intelligence':ti,ab,kw OR 'wppsi':ti,ab,kw OR 'binet test':ti,ab,kw OR 'developmental psychology':ti,ab,kw OR 'academic success':ti,ab,kw OR 'academic achievement':ti,ab,kw OR 'education success':ti,ab,kw OR 'education achievement':ti,ab,kw OR 'academic performance':ti,ab,kw OR 'academic test score':ti,ab,kw OR 'educational performance':ti,ab,kw OR 'educational test score':ti,ab,kw OR 'educational test performance':ti,ab,kw OR 'learning curve':ti,ab,kw OR 'psychomotor performance':ti,ab,kw OR 'visual motor performance':ti,ab,kw OR 'visuomotor coordination':ti,ab,kw OR 'perceptual motor performance':ti,ab,kw OR 'aptitude test':ti,ab,kw OR 'multitasking behavior':ti,ab,kw OR 'underachievement':ti,ab,kw OR 'learning':ti,ab,kw OR 'learn':ti,ab,kw OR 'learnings':ti,ab,kw OR 'learns':ti,ab,kw OR 'problem solving':ti,ab,kw OR 'thinking':ti,ab,kw) AND ('child':ti,ab,kw OR 'children':ti,ab,kw OR 'childrens':ti,ab,kw OR 'childs':ti,ab,kw OR 'boy':ti,ab,kw OR 'boys':ti,ab,kw OR 'boyhood':ti,ab,kw OR 'girl':ti,ab,kw OR 'girls':ti,ab,kw OR 'girlhood':ti,ab,kw) AND ('iron':ti,ab,kw OR 'ferric':ti,ab,kw OR 'ferrous':ti,ab,kw OR 'fe':ti,ab,kw OR 'anemia':ti,ab,kw OR 'anaemia':ti,ab,kw OR 'iron-deficiency':ti,ab,kw OR 'dietary supplement':ti,ab,kw OR 'diet supplement':ti,ab,kw OR 'minerals supplement':ti,ab,kw OR 'mineral supplement':ti,ab,kw OR 'multinutrient supplement':ti,ab,kw OR 'multinutrients supplement':ti,ab,kw OR 'micronutrient supplement':ti,ab,kw OR 'trace element supplement':ti,ab,kw) AND ([systematic review]/lim OR [meta analysis]/lim) | 163 |
| Scielo | ("cognitive development") AND ("iron supplementation") AND ("children") AND ("systematic review" OR "meta-analysis") | 0 |
| Google Scholar | ("cognitive development") + ("iron supplementation" OR "mineral supplement" OR "multinutrient supplement" OR "micronutrient supplement") + ("child" OR "children") + ("systematic review" OR "meta-analysis") | 100 |
| Proquest Dissertations and Theses | ("cognitive development") AND ("iron supplementation") AND ("children") AND ("systematic review" OR "meta-analysis") NOT (“Literature” OR “cross-sectional” OR “narrative”) | 76 |
| OpenGrey | ("cognitive development") AND ("iron supplementation") AND ("children") AND ("systematic review" OR "meta-analysis") | 0 |
