# Supplementary material for: Effect of iron supplements on cognitive development in children: an umbrella review
Source: Front Nutr. 2026 Feb 3;13:1718507. doi: 10.3389/fnut.2026.1718507 (PMC12909201; doi:10.3389/fnut.2026.1718507)
Supplement: Supplementary file 2 [file Table_2.docx]

Supplementary Material 2. Reason for exclusion of studies

| **Author** | **Reason for exclusion** |
| --- | --- |
| O'Connor et al. (1) | No iron supplement |
| O'Connor et al. (2) |  |

**References:**

1. O’Connor LE, Spill MK, Saha S, Balalian A, Davis JS, MacFarlane AJ. Seafood and Neurocognitive Development in Children: A Systematic Review. *Adv Nutr* (2025) 16:100391. doi: 10.1016/j.advnut.2025.100391

2. O’Connor LE, Spill MK, Saha S, Balalian AA, Davis JS, MacFarlane AJ. Seafood During Pregnancy and Lactation and Child Neurocognitive Development: A Systematic Review. *Adv Nutr* (2025) 16:100414. doi: 10.1016/j.advnut.2025.100414
