# Supplementary material for: Effect of iron supplements on cognitive development in children: an umbrella review
Source: Front Nutr. 2026 Feb 3;13:1718507. doi: 10.3389/fnut.2026.1718507 (PMC12909201; doi:10.3389/fnut.2026.1718507)
Supplement: Supplementary file 3 [file Table_3.docx]

Supplementary Material 3. Characteristics of included studies

| **Authors** | **Year** | **Study design** | **Country** | **Included study design** | **Number of studies (qualitative / quantitative)** | **Register** | **PRISMA** | **GRADE** | **ROBIS** | **Outcomes** | | | **Conclusions** |
| --- | --- | --- | --- | --- | --- | --- | --- | --- | --- | --- | --- | --- | --- |
| Tian et al. (1) | 2025 | SR with MA | China | RCTs | 8/8 | Yes | Yes | Yes | Low | Mental development | MD = -0.93 (-3.87 – 2.02) | Iron supplementation improves hematological parameters and reduces IDA in the short term (6 months) in healthy infants fed exclusively with breast milk, but does not maintain these benefits at 12 months. Furthermore, it may delay growth (weight and head circumference). | |
| Moumin et al. (2) | 2024 | SR | Australia | RCTs | 5/0 | Yes | Yes | Yes | Low | Higher doses of prenatal iron, compared with lower doses, neither benefited nor harmed cognitive development in infants younger than 12 months. | | | Current evidence does not strongly support a clear benefit of prenatal iron supplementation on child neurodevelopment in high- or upper-middle-income countries. |
| Gutema et al. (3) | 2023 | SR with MA | Ethiopia and Belgium | RCTs | 13/12 | Yes | Yes | No | Low | Intelligence | SMD = 0.46 (0.19 – 0.73) | Iron supplementation may improve cognitive development in schoolchildren with iron deficiency, but there is no clear evidence of this benefit in children without deficiency. | |
|  |  |  |  |  |  |  |  |  |  | Intelligence – Fe only | SMD = 0.2 (-0.04 – 0.44) |  |  |
|  |  |  |  |  |  |  |  |  |  | Intelligence – Anemic | SMD = 0.79 (0.41 – 1.16) |  |  |
|  |  |  |  |  |  |  |  |  |  | Intelligence – Non-anemic | SMD = 0.01 (-0.1 – 0.12) |  |  |
|  |  |  |  |  |  |  |  |  |  | Attention and concentration | SMD = 0.45 (0.09 – 0.8) |  |  |
|  |  |  |  |  |  |  |  |  |  | Attention and concentration – Fe only | SMD = 0.33 (-0.39 – 1.05) |  |  |
|  |  |  |  |  |  |  |  |  |  | Memory | SMD = 0.45 (0.04 – 0.69) |  |  |
|  |  |  |  |  |  |  |  |  |  | Memory – Fe only | SMD = 0.33 (0.08 – 0.57) |  |  |
|  |  |  |  |  |  |  |  |  |  | Memory – Anemic | SMD = 0.47 (0.13 – 0.81) |  |  |
|  |  |  |  |  |  |  |  |  |  | Memory – Non-anemic | SMD = -0.02 (-1.01 – 0.97) |  |  |
|  |  |  |  |  |  |  |  |  |  | School archievement | SMD = 0.0 (-0.21 – 0.21) |  |  |
|  |  |  |  |  |  |  |  |  |  | School archievement – Anemic | SMD = -0.12 (-0.63 – 0.39) |  |  |
|  |  |  |  |  |  |  |  |  |  | School archievement – Non-anemic | SMD = 0.43 (-0.74 – 1.59) |  |  |
| Mutua et al. (4) | 2021 | SR with MA | Kenya and United Kigdom | RCTs | 35/7 | Yes | Yes | No | Low | Cognitive development | MD = 1.73 (-1.05 – 4.52) | There is no evidence on the effects of iron supplementation or fortification on neurobehavioral outcomes in children. | |
|  |  |  |  |  |  |  |  |  |  | Cognitive development – Anemic | MD = -7.2 (-21.58 – 7.18) |  |  |
| McCann et al. (5) | 2020 | SR | United Kigdom and Gambia | RCTs | 28/0 | Yes | Yes | No | Low | No improvement was found between iron supplementation and cognitive development in children aged 0–24 months. However, an improvement was reported in children aged 6–59 months. | | The impact of iron supplementation on early development is inconsistent. | |
| Ip et al. (6) | 2017 | SR with MA | China | RCTs and OS | 48/41 | No | Yes | No | High | Cognitive development | SMD = 0.09 (0.03 – 0.15) | Infant iron supplementation was beneficial for cognitive development, but could be optimized by providing multiple nutrients. | |
| Cai et al. (7) | 2017 | SR with MA | Canada | RCTs | 4/4 | Yes | No | No | Unclear | Mental development | MD = 0.0 (-5.5 – 5.5) | Iron supplementation in healthy infants fed exclusively with breast milk has no effect on cognitive development. | |
| Petry et al. (8) | 2016 | SR with MA | Switzerland and United State | RCTs and Q-Es | 90/85 | No | No | Yes | Unclear | Mental development | MD = 0.4 (-0.9 – 1.7) | Providing iron in relatively low daily doses to young children does not have an effect on their mental development. | |
| Guo et al. (9) | 2015 | SR with MA | China | RCTs | 5/5 | No | No | No | High | Cognitive development | MD = 1.05 (-2.69 – 4.79) | Iron supplementation does not improve overall cognitive outcomes in primary school children. | |
| Thompson et al. (10) | 2013 | SR with MA | Australia | RCTs and Q-Es | 15/9 | Yes | No | Yes | Low | Cognitive development | MD = 0.25 (0.06 – 0.45) | Daily iron supplementation increases cognitive development. | |
| Low et al. (11) | 2013 | SR with MA | Australia | RCTs | 32/28 | Yes | No | No | Unclear | Cognitive development | SMD = 0.5 (0.11 – 0.9) | Iron supplementation benefits overall cognitive performance. | |
|  |  |  |  |  |  |  |  |  |  | Cognitive development – Anemic | SMD = 0.29 (0.07 – 0.51) |  |  |
|  |  |  |  |  |  |  |  |  |  | Cognitive development – Non-anemic | SMD = 0.01 (-0.1 – 0.11) |  |  |
|  |  |  |  |  |  |  |  |  |  | Intelligence | MD = 4.58 (-2.5 – 11.66) |  |  |
|  |  |  |  |  |  |  |  |  |  | Intelligence – Anemic | MD = 4.55 (0.16 – 8.94) |  |  |
|  |  |  |  |  |  |  |  |  |  | Intelligence – Non-anemic | MD = 0.08 (-1.86 – 2.01) |  |  |
| Wang et al. (12) | 2013 | SR with MA | China | RCTs | 8/7 | No | No | Yes | Low | Mental development – Anemic | MD = 1.04 (-1.3 – 3.39) | There is no evidence that iron treatment in children has an effect on cognitive development in the 30 days following the start of therapy. | |
| Pasricha et al. (13) | 2013 | SR with MA | Australia | RCTs | 33/33 | Yes | No | No | Low | Mental development | MD = 1.65 (-0.63 – 3.94) | In children aged 4 to 23 months, daily iron supplementation on cognitive development is uncertain. | |
|  |  |  |  |  |  |  |  |  |  | Mental development – Anemic | MD = 4.46 (-9.32 – 18.24) |  |  |
|  |  |  |  |  |  |  |  |  |  | Mental development – Non-anemic | MD = 1.49 (-1.08 – 4.07) |  |  |
| Abdullah et al. (14) | 2013 | SR with MA | Canada | RCTs | 2/2 | No | No | No | Unclear | Mental development | MD = 4.14 (0.1 – 8.18) | Oral Fe therapy in children increases cognitive development. | |
| Hermoso et al. (15) | 2011 | SR | Germany, Serbia and Spain | RCTs | 14/0 | No | No | No | Unclear | An improvement was observed between iron supplementation and cognitive development in children. | | There is a moderate positive effect of iron supplementation on cognition in infants and children after supplementation periods of at least 2 months duration. | |
| Szajewska et al. (16) | 2010 | SR with MA | Poland | RCTs | 5/3 | No | No | No | Unclear | Mental development | MD = 1.66 (-0.14 – 3.47) | Iron supplementation in infants does not appear to alter their mental development. | |
| Sachdev et al. (17) | 2005 | SR with MA | India and United State | RCTs | 17/14 | No | No | No | Unclear | Mental development | SMD = 0.3 (0.15 – 0.46) | Iron supplementation modestly improves cognitive development in children. | |
|  |  |  |  |  |  |  |  |  |  | Mental development – Anemic | SMD = 0.5 (0.25 – 0.75) |  |  |
|  |  |  |  |  |  |  |  |  |  | Mental development – Non-anemic | SMD = -0.11 (-0.36 – 0.14) |  |  |

SR = Systematic review; MA = Meta-analysis; RCT = Randomized clinical trial; OS = Observational study; Q-E = Quasi-Experimental study; MD = Mean difference; SMD = Standard mean difference; Fe = Iron; IDA = Iron deficiency anemia

**References**

1. Tian K, Liu W, Huang Y, Zhou R, Wang Y. Effect of iron supplementation in healthy exclusively breastfed infants: a systematic review and meta-analysis. *Front Pediatr* (2025) 13:1587457. doi: 10.3389/fped.2025.1587457

2. Moumin NA, Shepherd E, Liu K, Makrides M, Gould JF, Green TJ, Grzeskowiak LE. The Effects of Prenatal Iron Supplementation on Offspring Neurodevelopment in Upper Middle- or High-Income Countries: A Systematic Review. *Nutrients* (2024) 16:2499. doi: 10.3390/nu16152499

3. Gutema BT, Sorrie MB, Megersa ND, Yesera GE, Yeshitila YG, Pauwels NS, De Henauw S, Abbeddou S. Effects of iron supplementation on cognitive development in school-age children: Systematic review and meta-analysis. *PLoS One* (2023) 18:e0287703. doi: 10.1371/journal.pone.0287703

4. Mutua AM, Mwangi K, Abubakar A, Atkinson SH. Effects of iron intake on neurobehavioural outcomes in African children: a systematic review and meta-analysis of randomised controlled trials. *Wellcome Open Res* (2021) 6:181. doi: 10.12688/wellcomeopenres.16931.2

5. McCann S, Amadó MP, Moore SE. The role of iron in brain development: A systematic review. *Nutrients* (2020) 12:1–23. doi: 10.3390/nu12072001

6. Ip P, Ho FKW, Rao N, Sun J, Young ME, Chow CB, Tso W, Hon KL. Impact of nutritional supplements on cognitive development of children in developing countries: A meta-analysis. *Sci Rep* (2017) 7:10611. doi: 10.1038/s41598-017-11023-4

7. Cai C, Granger M, Eck P, Friel J. Effect of Daily Iron Supplementation in Healthy Exclusively Breastfed Infants: A Systematic Review with Meta-Analysis. *Breastfeeding Med* (2017) 12:597–603. doi: 10.1089/bfm.2017.0003

8. Petry N, Olofin I, Boy E, Donahue Angel MD, Rohner F. The effect of low dose Iron and zinc intake on child micronutrient status and development during the first 1000 days of life: A systematic review and meta-analysis. *Nutrients* (2016) 8: doi: 10.3390/nu8120773

9. Guo X-M, Liu H, Qian J. Daily iron supplementation on cognitive performance in primary-school-aged children with and without anemia: a meta-analysis. *Int J Clin Exp Med* (2015) 8:16107–16111.

10. Thompson J, Biggs B-A, Pasricha S-R. Effects of daily iron supplementation in 2- to 5-year-old children: systematic review and meta-analysis. *Pediatrics* (2013) 131:739–753. doi: 10.1542/peds.2012-2256

11. Low M, Farrell A, Biggs B-A, Pasricha S-R. Effects of daily iron supplementation in primary-school–aged children: systematic review and meta-analysis of randomized controlled trials. *CMAJ* (2013) 185:E791–E802. doi: 10.1503/cmaj.130628

12. Wang B, Zhan S, Gong T, Lee L. Iron therapy for improving psychomotor development and cognitive function in children under the age of three with iron deficiency anaemia. *Cochrane Database Syst Rev* (2013) 2013:CD001444. doi: 10.1002/14651858.CD001444.pub2

13. Pasricha S-R, Hayes E, Kalumba K, Biggs B-A. Effect of daily iron supplementation on health in children aged 4-23 months: a systematic review and meta-analysis of randomised controlled trials. *Lancet Glob Health* (2013) 1:e77–e86. doi: 10.1016/S2214-109X(13)70046-9

14. Abdullah K, Kendzerska T, Shah P, Uleryk E, Parkin PC. Efficacy of oral iron therapy in improving the developmental outcome of pre-school children with non-anaemic iron deficiency: A systematic review. *Public Health Nutr* (2013) 16:1497–1506. doi: 10.1017/S1368980012003709

15. Hermoso M, Vucic V, Vollhardt C, Arsic A, Roman-Viñas B, Iglesia-Altaba I, Gurinovic M, Koletzko B. The effect of iron on cognitive development and function in infants, children and adolescents: a systematic review. *Ann Nutr Metab* (2011) 59:154–165. doi: 10.1159/000334490

16. Szajewska H, Ruszczynski M, Chmielewska A. Effects of iron supplementation in nonanemic pregnant women, infants, and young children on the mental performance and psychomotor development of children: a systematic review of randomized controlled trials. *Am J Clin Nutr* (2010) 91:1684–1690. doi: 10.3945/ajcn.2010.29191

17. Sachdev H, Gera T, Nestel P. Effect of iron supplementation on mental and motor development in children: systematic review of randomised controlled trials. *Public Health Nutr* (2005) 8:117–132. doi: 10.1079/phn2004677
