# Supplementary material for: Effect of iron supplements on cognitive development in children: an umbrella review
Source: Front Nutr. 2026 Feb 3;13:1718507. doi: 10.3389/fnut.2026.1718507 (PMC12909201; doi:10.3389/fnut.2026.1718507)
Supplement: Supplementary file 4 [file Table_4.docx]

Supplementary Material 4. Assessment of the methodological quality and the quality of the evidence of the included studies

| Authors | Year | AMSTAR – 2 | | | | | | | | | | | | | | | | Overall confidence |
| --- | --- | --- | --- | --- | --- | --- | --- | --- | --- | --- | --- | --- | --- | --- | --- | --- | --- | --- |
|  |  | 1 | 2* | 3 | 4* | 5 | 6 | 7* | 8 | 9* | 10 | 11* | 12 | 13* | 14 | 15* | 16 |  |
| Tian et al. (1) | 2025 | Yes | Yes | Yes | Yes | Yes | Yes | Yes | Yes | Yes | No | Yes | Yes | Yes | Yes | Yes | Yes | High |
| Moumin et al. (2) | 2024 | Yes | Yes | Yes | Yes | Yes | Yes | Yes | Yes | Yes | No | No meta-analysis | | Yes | Yes | No meta-analysis | Yes | High |
| Gutema et al. (3) | 2023 | Yes | Yes | Yes | Yes | Yes | Yes | Yes | Yes | Yes | No | No | Yes | Yes | Yes | Yes | Yes | Low |
| Mutua et al. (4) | 2021 | Yes | Yes | Yes | Yes | Yes | Yes | Yes | Yes | Yes | No | Yes | Yes | Yes | Yes | No | Yes | Low |
| McCann et al. (5) | 2020 | Yes | Yes | Yes | Yes | Yes | Yes | Yes | Yes | Yes partial | No | No meta-analysis | | Yes | Yes | No meta-analysis | Yes | High |
| Ip et al. (6) | 2017 | Yes | No | Yes | Yes | No | No | Yes | Yes | No | No | Yes | Yes | Yes | Yes | Yes | Yes | Critically low |
| Cai et al. (7) | 2017 | Yes | Yes | Yes | Yes | No | No | Yes | Yes | Yes | No | Yes | Yes | No | No | No | Yes | Critically low |
| Petry et al. (8) | 2016 | Yes | No | Yes | Yes | No | No | Yes | Yes | Yes partial | No | Yes | Yes | Yes | Yes | Yes | Yes | Low |
| Guo et al. (9) | 2015 | Yes | No | Yes | No | Yes | Yes | No | Yes | No | No | Yes | Yes | Yes | Yes | Yes | Yes | Critically low |
| Thompson et al. (10) | 2013 | Yes | Yes | Yes | Yes | Yes | Yes | Yes | Yes | Yes | No | No | Yes | Yes | Yes | No | Yes | Critically low |
| Low et al. (11) | 2013 | Yes | Yes | Yes | Yes | Yes | Yes | Yes | Yes | Yes | No | No | Yes | Yes | Yes | Yes | Yes | Low |
| Wang et al. (12) | 2013 | Yes | No | Yes | Yes | Yes | Yes | Yes | Yes | Yes | No | Yes | Yes | Yes | Yes | Yes | Yes | Low |
| Pasricha et al. (13) | 2013 | Yes | No | Yes | Yes | Yes | Yes | Yes | Yes | Yes | No | Yes | Yes | Yes | Yes | Yes | Yes | Low |
| Abdullah et al. (14) | 2013 | Yes | No | Yes | Yes | Yes | Yes | Yes | Yes | Yes | No | Yes | Yes | Yes | Yes | Yes | Yes | Low |
| Hermoso et al. (15) | 2011 | Yes | No | Yes | Yes | Yes | Yes | Yes | Yes | Yes partial | No | No meta-analysis | | Yes | Yes | No meta-analysis | Yes | Low |
| Szajewska et al. (16) | 2010 | Yes | No | Yes | Yes | Yes | Yes | Yes | Yes | Yes partial | No | No | Yes | Yes | Yes | Yes | Yes | Critically low |
| Sachdev et al. (17) | 2005 | Yes | No | Yes | Yes | No | No | Yes | Yes | Yes partial | No | No | Yes | Yes | Yes | Yes | Yes | Critically low |

AMSTAR = A MeaSurement Tool to Assess Systemic Reviews

1 = Did the research questions and inclusion criteria for the review include the components of PICO?

2 = Did the report of the review contain an explicit statement that the review methods were established prior to the conduct of the review and did the report justify any significant deviations from the protocol?

3 = Did the review authors explain their selection of the study designs for inclusion in the review?

4 = Did the review authors use a comprehensive literature search strategy?

5 = Did the review authors perform study selection in duplicate?

6 = Did the review authors perform data extraction in duplicate?

7 = Did the review authors provide a list of excluded studies and justify the exclusions?

8 = Did the review authors describe the included studies in adequate detail?

9 = Did the review authors use a satisfactory technique for assessing the risk of bias (RoB) in individual studies that were included in the review?

10 = Did the review authors report on the sources of funding for the studies included in the review?

11 = If meta-analysis was performed, did the review authors use appropriate methods for statistical combination of results?

12 = If meta-analysis was performed, did the review authors assess the potential impact of RoB in individual studies on the results of the meta-analysis or other evidence synthesis?

13 = Did the review authors account for RoB in primary studies when interpreting/discussing the results of the review?

14 = Did the review authors provide a satisfactory explanation for, and discussion of, any heterogeneity observed in the results of the review?

15 = If they performed quantitative synthesis did the review authors carry out an adequate investigation of publication bias (small study bias) and discuss its likely impact on the results of the review?

16 = Did the review authors report any potential sources of conflict of interest, including any funding they received for conducting the review?

* = Critical domain

**References**

1. Tian K, Liu W, Huang Y, Zhou R, Wang Y. Effect of iron supplementation in healthy exclusively breastfed infants: a systematic review and meta-analysis. *Front Pediatr* (2025) 13:1587457. doi: 10.3389/fped.2025.1587457

2. Moumin NA, Shepherd E, Liu K, Makrides M, Gould JF, Green TJ, Grzeskowiak LE. The Effects of Prenatal Iron Supplementation on Offspring Neurodevelopment in Upper Middle- or High-Income Countries: A Systematic Review. *Nutrients* (2024) 16:2499. doi: 10.3390/nu16152499

3. Gutema BT, Sorrie MB, Megersa ND, Yesera GE, Yeshitila YG, Pauwels NS, De Henauw S, Abbeddou S. Effects of iron supplementation on cognitive development in school-age children: Systematic review and meta-analysis. *PLoS One* (2023) 18:e0287703. doi: 10.1371/journal.pone.0287703

4. Mutua AM, Mwangi K, Abubakar A, Atkinson SH. Effects of iron intake on neurobehavioural outcomes in African children: a systematic review and meta-analysis of randomised controlled trials. *Wellcome Open Res* (2021) 6:181. doi: 10.12688/wellcomeopenres.16931.2

5. McCann S, Amadó MP, Moore SE. The role of iron in brain development: A systematic review. *Nutrients* (2020) 12:1–23. doi: 10.3390/nu12072001

6. Ip P, Ho FKW, Rao N, Sun J, Young ME, Chow CB, Tso W, Hon KL. Impact of nutritional supplements on cognitive development of children in developing countries: A meta-analysis. *Sci Rep* (2017) 7:10611. doi: 10.1038/s41598-017-11023-4

7. Cai C, Granger M, Eck P, Friel J. Effect of Daily Iron Supplementation in Healthy Exclusively Breastfed Infants: A Systematic Review with Meta-Analysis. *Breastfeeding Med* (2017) 12:597–603. doi: 10.1089/bfm.2017.0003

8. Petry N, Olofin I, Boy E, Donahue Angel MD, Rohner F. The effect of low dose Iron and zinc intake on child micronutrient status and development during the first 1000 days of life: A systematic review and meta-analysis. *Nutrients* (2016) 8: doi: 10.3390/nu8120773

9. Guo X-M, Liu H, Qian J. Daily iron supplementation on cognitive performance in primary-school-aged children with and without anemia: a meta-analysis. *Int J Clin Exp Med* (2015) 8:16107–16111.

10. Thompson J, Biggs B-A, Pasricha S-R. Effects of daily iron supplementation in 2- to 5-year-old children: systematic review and meta-analysis. *Pediatrics* (2013) 131:739–753. doi: 10.1542/peds.2012-2256

11. Low M, Farrell A, Biggs B-A, Pasricha S-R. Effects of daily iron supplementation in primary-school–aged children: systematic review and meta-analysis of randomized controlled trials. *CMAJ* (2013) 185:E791–E802. doi: 10.1503/cmaj.130628

12. Wang B, Zhan S, Gong T, Lee L. Iron therapy for improving psychomotor development and cognitive function in children under the age of three with iron deficiency anaemia. *Cochrane Database Syst Rev* (2013) 2013:CD001444. doi: 10.1002/14651858.CD001444.pub2

13. Pasricha S-R, Hayes E, Kalumba K, Biggs B-A. Effect of daily iron supplementation on health in children aged 4-23 months: a systematic review and meta-analysis of randomised controlled trials. *Lancet Glob Health* (2013) 1:e77–e86. doi: 10.1016/S2214-109X(13)70046-9

14. Abdullah K, Kendzerska T, Shah P, Uleryk E, Parkin PC. Efficacy of oral iron therapy in improving the developmental outcome of pre-school children with non-anaemic iron deficiency: A systematic review. *Public Health Nutr* (2013) 16:1497–1506. doi: 10.1017/S1368980012003709

15. Hermoso M, Vucic V, Vollhardt C, Arsic A, Roman-Viñas B, Iglesia-Altaba I, Gurinovic M, Koletzko B. The effect of iron on cognitive development and function in infants, children and adolescents: a systematic review. *Ann Nutr Metab* (2011) 59:154–165. doi: 10.1159/000334490

16. Szajewska H, Ruszczynski M, Chmielewska A. Effects of iron supplementation in nonanemic pregnant women, infants, and young children on the mental performance and psychomotor development of children: a systematic review of randomized controlled trials. *Am J Clin Nutr* (2010) 91:1684–1690. doi: 10.3945/ajcn.2010.29191

17. Sachdev H, Gera T, Nestel P. Effect of iron supplementation on mental and motor development in children: systematic review of randomised controlled trials. *Public Health Nutr* (2005) 8:117–132. doi: 10.1079/phn2004677
