# Supplementary material for: Effect of iron supplements on cognitive development in children: an umbrella review
Source: Front Nutr. 2026 Feb 3;13:1718507. doi: 10.3389/fnut.2026.1718507 (PMC12909201; doi:10.3389/fnut.2026.1718507)
Supplement: Supplementary file 5 [file Table_5.docx]

Supplementary Material 5. Overlapping of primary studies in systematic reviews

| **Primary studies** | **Systematic reviews that included the primary studies** | **Times that primary studies were included** |
| --- | --- | --- |
| Idjradinata et al. (1) | Mutua et al. (2), McCann et al. (3), Ip et al. (4), Pasricha et al. (5), Abdullah et al. (6), Hermoso et al. (7), Sachdev et al. (8) | 7 |
| Lind et al. (9) | Mutua et al. (2), McCann et al. (3), Ip et al. (4), Pasricha et al. (5), Szajewska et al. (10) | 5 |
| Lozoff et al. (11) | Mutua et al. (2), McCann et al. (3), Pasricha et al. (5), Sachdev et al. (8), Wang et al. (12) | 5 |
| Friel et al. (13) | McCann et al. (3), Szajewska et al. (10), Tian et al. (14), Cai et al. (15) | 4 |
| Akman et al. (16) | Mutua et al. (2), McCann et al. (3), Pasricha et al. (5), Abdullah et al. (6), | 4 |
| Moffatt et al. (17) | McCann et al. (3), Sachdev et al. (8), Szajewska et al. (10), Petry et al. (18) | 4 |
| Soemantri et al. (19) | Sachdev et al. (8), Gutema et al. (20), Guo et al. (21), Low et al. (22) | 4 |
| Walter et al. (23) | McCann et al. (3), Pasricha et al. (5), Sachdev et al. (8), Wang et al. (12) | 4 |
| Oski et al. (24) | Mutua et al. (2), McCann et al. (3), Sachdev et al. (8) | 3 |
| Morley et al. (25) | McCann et al. (3), Sachdev et al. (8), Petry et al. (18) | 3 |
| Yalçin et al. (26) | Mutua et al. (2), Pasricha et al. (5), Petry et al. (18) | 3 |
| Seshadri et al. (27) | Sachdev et al. (8), Guo et al. (21), Low et al. (22) | 3 |
| Pollitt et al. (28) | Sachdev et al. (8), Gutema et al. (20), Low et al. (22) | 3 |
| Black et al. (29) | Mutua et al. (2), McCann et al. (3) | 2 |
| Lozoff et al. (30) | McCann et al. (3), Ip et al. (4) | 2 |
| Gopaldas et al. (31) | Gutema et al. (20), Low et al. (22) | 2 |
| Kashyap et al. (32) | Gutema et al. (20), Low et al. (22) | 2 |
| Seshadri et al. (33) | Guo et al. (21), Low et al. (22) | 2 |
| Sungthong et al. (34) | Guo et al. (21), Low et al. (22) | 2 |
| Metallinos-Katsaras et al. (35) | McCann et al. (3), Hermoso et al. (7) | 2 |
| Ayoya et al. (36) | Gutema et al. (20), Low et al. (22) | 2 |
| Kimmons et al. (37) | Sachdev et al. (8), Wang et al. (12) | 2 |
| Driva et al. (38) | Sachdev et al. (8), Wang et al. (12) | 2 |
| Soemantri et al. (39) | Sachdev et al. (8), Gutema et al. (20) | 2 |
| Soewondo et al. (40) | Hermoso et al. (7), Sachdev et al. (8) | 2 |

**References**

1. Idjradinata P, Pollitt E. Reversal of developmental delays in iron-deficient anaemic infants treated with iron. *Lancet* (1993) 341:1–4. doi: 10.1016/0140-6736(93)92477-b

2. Mutua AM, Mwangi K, Abubakar A, Atkinson SH. Effects of iron intake on neurobehavioural outcomes in African children: a systematic review and meta-analysis of randomised controlled trials. *Wellcome Open Res* (2021) 6:181. doi: 10.12688/wellcomeopenres.16931.2

3. McCann S, Amadó MP, Moore SE. The role of iron in brain development: A systematic review. *Nutrients* (2020) 12:1–23. doi: 10.3390/nu12072001

4. Ip P, Ho FKW, Rao N, Sun J, Young ME, Chow CB, Tso W, Hon KL. Impact of nutritional supplements on cognitive development of children in developing countries: A meta-analysis. *Sci Rep* (2017) 7:10611. doi: 10.1038/s41598-017-11023-4

5. Pasricha S-R, Hayes E, Kalumba K, Biggs B-A. Effect of daily iron supplementation on health in children aged 4-23 months: a systematic review and meta-analysis of randomised controlled trials. *Lancet Glob Health* (2013) 1:e77–e86. doi: 10.1016/S2214-109X(13)70046-9

6. Abdullah K, Kendzerska T, Shah P, Uleryk E, Parkin PC. Efficacy of oral iron therapy in improving the developmental outcome of pre-school children with non-anaemic iron deficiency: A systematic review. *Public Health Nutr* (2013) 16:1497–1506. doi: 10.1017/S1368980012003709

7. Hermoso M, Vucic V, Vollhardt C, Arsic A, Roman-Viñas B, Iglesia-Altaba I, Gurinovic M, Koletzko B. The effect of iron on cognitive development and function in infants, children and adolescents: a systematic review. *Ann Nutr Metab* (2011) 59:154–165. doi: 10.1159/000334490

8. Sachdev H, Gera T, Nestel P. Effect of iron supplementation on mental and motor development in children: systematic review of randomised controlled trials. *Public Health Nutr* (2005) 8:117–132. doi: 10.1079/phn2004677

9. Lind T, Lönnerdal B, Stenlund H, Gamayanti IL, Ismail D, Seswandhana R, Persson L-A. A community-based randomized controlled trial of iron and zinc supplementation in Indonesian infants: effects on growth and development. *Am J Clin Nutr* (2004) 80:729–736. doi: 10.1093/ajcn/80.3.729

10. Szajewska H, Ruszczynski M, Chmielewska A. Effects of iron supplementation in nonanemic pregnant women, infants, and young children on the mental performance and psychomotor development of children: a systematic review of randomized controlled trials. *Am J Clin Nutr* (2010) 91:1684–1690. doi: 10.3945/ajcn.2010.29191

11. Lozoff B, Brittenham GM, Viteri FE, Wolf AW, Urrutia JJ. The effects of short-term oral iron therapy on developmental deficits in iron-deficient anemic infants. *J Pediatr* (1982) 100:351–357. doi: 10.1016/s0022-3476(82)80428-9

12. Wang B, Zhan S, Gong T, Lee L. Iron therapy for improving psychomotor development and cognitive function in children under the age of three with iron deficiency anaemia. *Cochrane Database Syst Rev* (2013) 2013:CD001444. doi: 10.1002/14651858.CD001444.pub2

13. Friel JK, Aziz K, Andrews WL, Harding SV, Courage ML, Adams RJ. A double-masked, randomized control trial of iron supplementation in early infancy in healthy term breast-fed infants. *J Pediatr* (2003) 143:582–586. doi: 10.1067/S0022-3476(03)00301-9

14. Tian K, Liu W, Huang Y, Zhou R, Wang Y. Effect of iron supplementation in healthy exclusively breastfed infants: a systematic review and meta-analysis. *Front Pediatr* (2025) 13:1587457. doi: 10.3389/fped.2025.1587457

15. Cai C, Granger M, Eck P, Friel J. Effect of Daily Iron Supplementation in Healthy Exclusively Breastfed Infants: A Systematic Review with Meta-Analysis. *Breastfeeding Med* (2017) 12:597–603. doi: 10.1089/bfm.2017.0003

16. Akman M, Cebeci D, Okur V, Angin H, Abali O, Akman AC. The effects of iron deficiency on infants’ developmental test performance. *Acta Paediatr* (2004) 93:1391–1396. doi: 10.1111/j.1651-2227.2004.tb02941.x

17. Moffatt ME, Longstaffe S, Besant J, Dureski C. Prevention of iron deficiency and psychomotor decline in high-risk infants through use of iron-fortified infant formula: a randomized clinical trial. *J Pediatr* (1994) 125:527–534. doi: 10.1016/s0022-3476(94)70003-6

18. Petry N, Olofin I, Boy E, Donahue Angel MD, Rohner F. The effect of low dose Iron and zinc intake on child micronutrient status and development during the first 1000 days of life: A systematic review and meta-analysis. *Nutrients* (2016) 8: doi: 10.3390/nu8120773

19. Soemantri AG. Preliminary findings on iron supplementation and learning achievement of rural Indonesian children. *Am J Clin Nutr* (1989) 50:698–701; discussion 701-702. doi: 10.1093/ajcn/50.3.689

20. Gutema BT, Sorrie MB, Megersa ND, Yesera GE, Yeshitila YG, Pauwels NS, De Henauw S, Abbeddou S. Effects of iron supplementation on cognitive development in school-age children: Systematic review and meta-analysis. *PLoS One* (2023) 18:e0287703. doi: 10.1371/journal.pone.0287703

21. Guo X-M, Liu H, Qian J. Daily iron supplementation on cognitive performance in primary-school-aged children with and without anemia: a meta-analysis. *Int J Clin Exp Med* (2015) 8:16107–16111.

22. Low M, Farrell A, Biggs B-A, Pasricha S-R. Effects of daily iron supplementation in primary-school–aged children: systematic review and meta-analysis of randomized controlled trials. *CMAJ* (2013) 185:E791–E802. doi: 10.1503/cmaj.130628

23. Walter T, De Andraca I, Chadud P, Perales CG. Iron deficiency anemia: adverse effects on infant psychomotor development. *Pediatrics* (1989) 84:7–17. doi: 10.1542/peds.84.1.7

24. Oski FA, Honig AS. The effects of therapy on the developmental scores of iron-deficient infants. *J Pediatr* (1978) 92:21–25. doi: 10.1016/s0022-3476(78)80063-8

25. Morley R, Abbott R, Fairweather-Tait S, MacFadyen U, Stephenson T, Lucas A. Iron fortified follow on formula from 9 to 18 months improves iron status but not development or growth: a randomised trial. *Arch Dis Child* (1999) 81:247–252. doi: 10.1136/adc.81.3.247

26. Yalçin SS, Yurdakök K, Açikgöz D, Ozmert E. Short-term developmental outcome of iron prophylaxis in infants. *Pediatr Int* (2000) 42:625–630. doi: 10.1046/j.1442-200x.2000.01299.x

27. Seshadri S, Gopaldas T. Impact of iron supplementation on cognitive functions in preschool and school-aged children: the Indian experience. *Am J Clin Nutr* (1989) 50:675–684; discussion 685-686. doi: 10.1093/ajcn/50.3.675

28. Pollitt E, Hathirat P, Kotchabhakdi NJ, Missell L, Valyasevi A. Iron deficiency and educational achievement in Thailand. *Am J Clin Nutr* (1989) 50:687–696; discussion 696-697. doi: 10.1093/ajcn/50.3.687

29. Black MM, Baqui AH, Zaman K, Ake Persson L, El Arifeen S, Le K, McNary SW, Parveen M, Hamadani JD, Black RE. Iron and zinc supplementation promote motor development and exploratory behavior among Bangladeshi infants. *Am J Clin Nutr* (2004) 80:903–910. doi: 10.1093/ajcn/80.4.903

30. Lozoff B, De Andraca I, Castillo M, Smith JB, Walter T, Pino P. Behavioral and developmental effects of preventing iron-deficiency anemia in healthy full-term infants. *Pediatrics* (2003) 112:846–854. doi: 10.1542/peds.112.4.846

31. Gopaldas T, Kale M, Bhardwaj P. Prophylactic iron supplementation for underprivileged school boys. II. Impact on selected tests of cognitive function. *Indian Pediatr* (1985) 22:737–743.

32. Kashyap P, Gopaldas T. Impact of hematinic supplementation on cognitive function in underprivileged school girls (8–15 Yrs of age). *Nutrition Research* (1987) 7:1117–1126. doi: 10.1016/S0271-5317(87)80037-4

33. Seshadri S, Hirode K, Naik P, Malhotra S. Behavioural responses of young anaemic Indian children to iron-folic acid supplements. *Br J Nutr* (1982) 48:233–240. doi: 10.1079/bjn19820109

34. Sungthong R, Mo-Suwan L, Chongsuvivatwong V, Geater AF. Once weekly is superior to daily iron supplementation on height gain but not on hematological improvement among schoolchildren in Thailand. *J Nutr* (2002) 132:418–422. doi: 10.1093/jn/132.3.418

35. Metallinos-Katsaras E, Valassi-Adam E, Dewey KG, Lönnerdal B, Stamoulakatou A, Pollitt E. Effect of iron supplementation on cognition in Greek preschoolers. *Eur J Clin Nutr* (2004) 58:1532–1542. doi: 10.1038/sj.ejcn.1602005

36. Ayoya MA, Spiekermann-Brouwer GM, Traoré AK, Garza C. Effect on school attendance and performance of iron and multiple micronutrients as adjunct to drug treatment of Schistosoma-infected anemic schoolchildren. *Food Nutr Bull* (2012) 33:235–241. doi: 10.1177/156482651203300403

37. Short term effects of intramuscular iron on the behaviour of iron-deficient children: a clinical trial. (2000)

38. Driva A, Kafatos A, Solman M. Iron deficiency and the cognitive and psychomotor development of children: A pilot study with institutionalised children. *Early Child Development and Care* (1985) 22:73–82. doi: 10.1080/0300443850220106

39. Soemantri AG, Pollitt E, Kim I. Iron deficiency anemia and educational achievement. *Am J Clin Nutr* (1985) 42:1221–1228. doi: 10.1093/ajcn/42.6.1221

40. Soewondo S, Husaini M, Pollitt E. Effects of iron deficiency on attention and learning processes in preschool children: Bandung, Indonesia. *Am J Clin Nutr* (1989) 50:667–673; discussion 673-674. doi: 10.1093/ajcn/50.3.667
